# Assessing Physiological Stress Responses in Student Nurses Using Mixed Reality Training

**DOI:** 10.3390/s25103222

**Published:** 2025-05-20

**Authors:** Kamelia Sepanloo, Daniel Shevelev, Young-Jun Son, Shravan Aras, Janine E. Hinton

**Affiliations:** 1 Edwardson School of Industrial Engineering, Purdue University, West Lafayette, IN 47907, USA; ksepanlo@purdue.edu (K.S.); yjson@purdue.edu (Y.-J.S.); 2 School of Information Science, University of Arizona, Tucson, AZ 85721, USA; dshevelev@arizona.edu; 3 Center for Biomedical Informatics and Biostatistics, University of Arizona, Tucson, AZ 85721, USA; shravanaras@arizona.edu; 4 College of Nursing, University of Arizona, Tucson, AZ 85721, USA

**Keywords:** physiological measures analysis, wearable sensors, mixed reality, nursing

## Abstract

This study explores nursing students’ stress responses while they are being trained in a mixed reality (MR) setting that replicates highly stressful clinical scenarios. Using measurements of physiological indices such as heart rate, electrodermal activity, and skin temperature, the study assesses the level of stress when the students interact with digital patients whose vital signs and symptoms interact dynamically to respond to student inputs. The simulation consists of six segments, during which critical events like hypotension and hypoxia occur, and the patient’s condition changes based on the nurse’s clinical decisions. Machine learning algorithms were then used to analyze the nurse’s physiological data and to classify different levels of stress. Among the models tested, the Stacking Classifier demonstrated the highest classification accuracy of 96.4%, outperforming both Random Forest (96.18%) and Gradient Boosting (95.35%). The results showed clear patterns of stress during the simulation segments. Statistical analysis also found significant differences in stress responses and identified key physiological markers linked to each stress level. This pioneering study demonstrates the effectiveness of MR as a training tool for healthcare professionals in high-pressured scenarios and lays the groundwork for further studies on stress management, adaptive training procedures, and real-time detection and intervention in MR-based nursing training.

## 1. Introduction

Stress is a dominant concern in the nursing field, and it greatly impacts the general well-being of nurses and patient care quality. Various studies emphasized reducing the stress level among medical professionals. A study conducted by [1] focused on the psychological effect of stress experienced by medical personnel serving at the frontline amidst the COVID-19 pandemic. Loss of control, personal illness, and susceptibility to infection were among the variables the study recognized as significant sources of stress. Similarly, ref. [2] emphasized the need to examine the level of stress among nurses in acute care to be able to establish the stressors that can compromise the provision of quality patient care.

Stress levels were shown through research to impact the competency and work performance of nurses, specifically in ICU settings. The environment of the ICU and the emotional impact of working in areas with high levels of stress are contributing factors to the stress of nurses [3]. Workload was shown to be associated with physiological stress responses in nurses; hence, it is essential to eliminate work overload and have sufficient rest to prevent stress elevation [4]. Moreover, studies focusing on specific nursing departments reveal that stress levels can vary significantly between different specializations. Research shows that nurses working in internal medicine departments report higher stress levels than their surgical counterparts, suggesting that the work environment and patient characteristics play critical roles in stress experiences [5].

There is evidence that high levels of stress in nursing students are related to low academic performance and satisfaction. For instance, ref. [6] confirmed that increased levels of stress while learning online, particularly during the COVID-19 pandemic, led to decreased satisfaction and poorer academic performance in nursing students. Similarly, ref. [7] confirmed that common stressors included academic workload and clinical environment problems, which cumulatively impacted students’ learning experiences. This is also emphasized by [8], who confirmed that stressors in clinical education greatly influence students’ learning abilities and overall educational performance. In support of this, ref. [9] explored the perspectives of nursing educators and undergraduate nursing students engaging in mixed reality-based remote simulations. Their study highlighted that integrating mixed reality into nursing education not only enhanced students’ engagement but also exposed them to realistic clinical stressors, providing opportunities to develop stress management strategies in a safe learning environment.

Several recent studies emphasized the importance of utilizing physiological measures in occupational stress quantification among nurses. Some of the key physiological correlations such as heart rate, electrodermal activity, and skin temperature are crucial in ascertaining stress in nurses [10]. For instance, ref. [11] utilized wearable ECG devices to assess heart rate and HRV among nurses, establishing a correlation between these physiological metrics and subjective stress responses, thus reinforcing HRV’s applicability in occupational health assessments. Similarly, ref. [12] demonstrated the effectiveness of various wearable sensors, including EDA, in monitoring stress in intensive care unit (ICU) nurses in real-time. However, further studies are needed in taking advantage of physiological measures in continuous stress and fatigue monitoring in nursing due to the existence of research gaps [13].

Additionally, immersive virtual scenarios with real-time feedback have been applied in psychological stress management, with encouraging results in enhancing coping skills and self-efficacy among nurses [14]. Virtual reality (VR) training was found to elicit similar stress responses to realistic face-to-face scenarios, demonstrating the effectiveness of VR in simulating high-stress environments for stress monitoring [15]. Furthermore, ref. [16] conducted a systematic review of virtual reality simulation effectiveness in nursing and midwifery education with a focus on its superiority in enhancing students’ procedural knowledge. Similarly, ref. [17] reviewed the role of digitally assisted mindfulness interventions in improving self-regulation and sustaining mental health. Their systematic review emphasized that incorporating digital tools into mindfulness practices significantly improved individuals’ ability to manage stress, suggesting potential applications of such technologies in nursing education to enhance mental resilience.

The continuous monitoring of physiological parameters during immersive training sessions can give valuable feedback on the levels of stress in nurses and allow the tailoring of interventions to reduce occupational stress more effectively [18]. The findings from [19] indicate that while various technology-delivered interventions exist, the integration of MR into stress management programs tailored for nursing is still limited. Therefore, further research is needed to explore the potential of mixed reality environment in nursing education, particularly regarding stress management [20,21].

Therefore, this study conducted a novel experiment to assess the stress levels of nurse learners in a mixed reality environment that was designed to simulate actual healthcare scenarios. The learners were equipped with an Empatica E4 wristband (manufactured by Empatica Inc. based in Cambridge, Massachusetts) to capture the key indicators of heart rate, electrodermal activity, and skin temperature. These markers were continuously recorded as the learners interacted with digital patients through the segments of the simulation. In recording real-time physiological data, the goal was to determine the change in stress level throughout the simulation and how it impacted clinical performance.

## 2. Experiment Design

**Figure 1:**
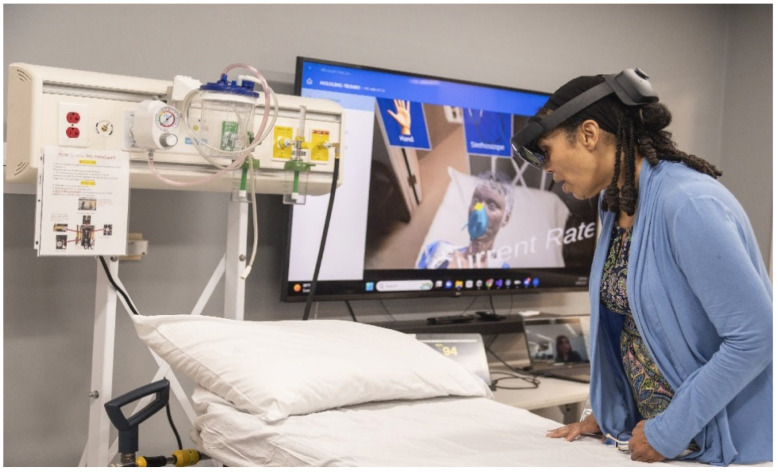
Learner engaged in the experiment, interacting with the digital patient and equipment within a physical environment.

**Figure 2:**
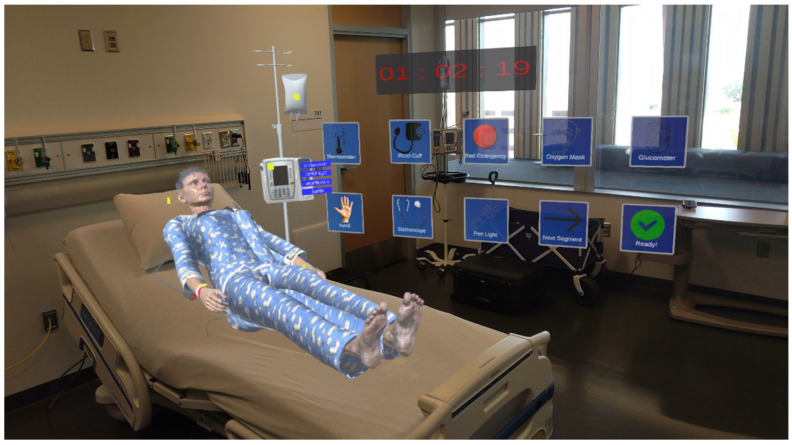
View inside of the HoloLens Headset.

The experiment was facilitated using a Microsoft HoloLens 2 MR headset (manufactured by Microsoft Inc., Redmond, WA, USA). All the digital patients and medical equipment were developed using the Unity 3D game engine (version 2022.3.19). Figure 1 shows a learner using the system, and Figure 2 shows what they viewed inside the headset.

Through the headset, the learner experienced a blend of digital and real elements, with digital patients and medical equipment overlaid onto the real-world objects. The digital patients were also equipped with an advanced conversational artificial intelligence (AI) model that allowed them to communicate naturally with the learner during the simulation. This model was developed using MindMeld conversational AI platform in Python 3.11.7, and could respond to the learner’s questions, actions, and remarks, simulating the interaction in a more realist way as a natural conversation with a patient. This contributed to realism, mimicking what nurses normally encounter in real life. The patient’s facial expressions also changed based on the health condition, responding to what learner has intervened and therefore made the whole experience more realistic [22].

Each training session took a duration of approximately 2 h per learner. At the beginning, the learners were given a tutorial to become familiar with the system and learn to use the digital equipment. Upon its completion, they were then introduced to an overview of the patient’s situation, medical history, physical findings, nursing recommendations, and orders from the physician. After preparing, the learners started the session and worked with the digital patient. They controlled the pace of simulation by pressing a “Next Segment” button, moving the scenario 2 h ahead, for a total of six segments. Depending on the learners’ actions and decisions, the status of the patient may improve or degrade. However, the patient would encounter hypotension and hypoxia at some point, simulating a medical emergency. At the end of the experiment, the learners were given a set of debriefing questions where they stated their objectives, patients’ needs, risks, and interventions during the simulation.

The main goal of the experiment was to measure the learner’s stress and perform analysis of features with respect to it while using the MR system. To that end, the physiological signals of the learners were captured with the Empatica E4 wearable sensor, which tracks heart rate, electrodermal activity, and skin temperature. As an initial step for the training, the physiological data of learners were recorded for 10 min to identify each learner’s pre-simulation initial stress level. This baseline measurement was taken as a reference basis for measuring any changes in stress during the simulation.

## 3. Methodology

### 3.1. Pre-Processing and Feature Extraction

**Figure 3:**
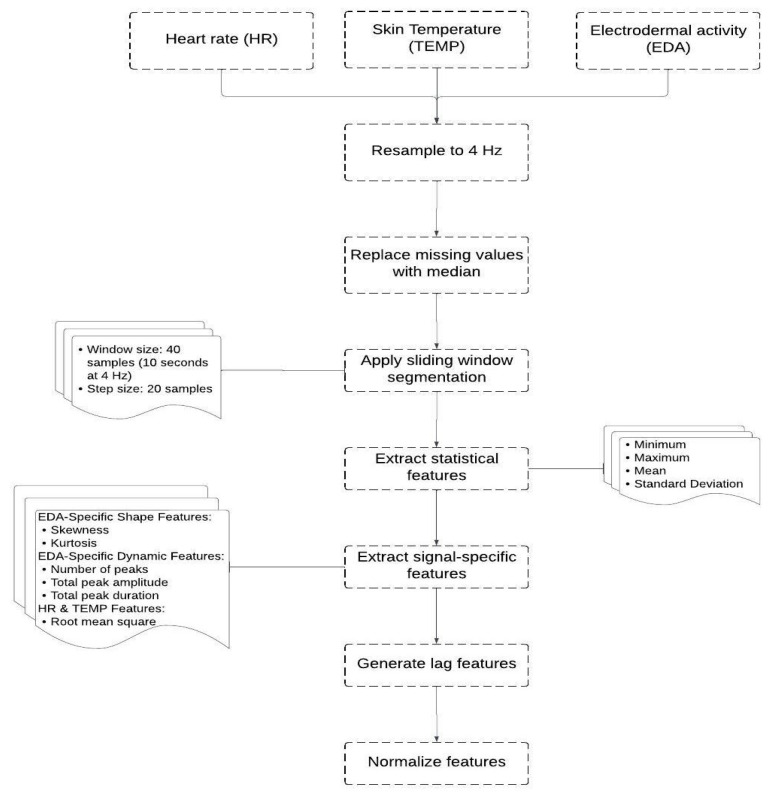
Steps of the data preprocessing.

Pre-processing is a crucial step in working with physiological data as it enables us to denoise the data, deal with missing values and prepare the data for proper analysis and modeling. For this purpose, a Python script was developed to preprocess the data, select significant features and prepare it for machine learning. Some of the key libraries utilized in the script are “pandas” for data manipulation and data arrangement, “numpy” for calculation operations, and “scipy” for statistical functionality. Figure 3 demonstrates all the steps followed in this pre-processing.

The process began with using each learner’s Heart Rate (HR), Skin Temperature (TEMP), and Electrodermal Activity (EDA) data. Since these signals have different sampling rates, they were first resampled to a unified rate of 4 Hz to ensure consistent data alignment and reduce information loss. Next, the data were cleaned to ensure that high-quality data were used in the model. A small percentage (1.02%) of missing values were present in the dataset. Therefore, missing values in each signal (EDA, HR, and TEMP) were replaced with the median of the respective feature, ensuring that data imputation did not distort the overall signal characteristics.

To extract relevant features, a sliding window technique was employed with a window size of 40 samples and a step size of 20 samples (50% overlap). Within each window, we computed statistical descriptors (minimum, maximum, mean, and standard deviation) for EDA, HR, and TEMP. For the EDA signal, additional shape-related features (skewness and kurtosis) were calculated to capture asymmetry and tailedness. Furthermore, EDA-specific dynamic features were derived using peak analysis, including the number of peaks (indicative of phasic responses), total peak amplitude, and total peak duration (both representing the intensity and temporal extent of EDA fluctuations).

The HR and TEMP signals had their root mean square (RMS) of first differences computed to quantify variability and short-term fluctuations within the window. In total, this resulted in 18 features describing the windowed physiological activity.

To account for temporal dependencies and trends in physiological responses, lagged features were generated. Specifically, the mean values of EDA, HR, and TEMP over the previous 1 to 10 windows were concatenated to the current feature vector. This yielded 30 additional lag-based features, which, combined with the 18 current-window features, formed a comprehensive feature set of 48 dimensions.

Prior to training the machine learning models, all features were normalized using min-max scaling to rescale their values into the [0, 1] range. Although min-max scaling can be sensitive to outliers, it was appropriate in this context because the windowed features exhibited stable ranges, and no extreme outliers were present after preprocessing.

### 3.2. Stress Detection Models

The models in this study were trained using the AffectiveROAD dataset [23], which was specifically designed to collect a broad set of physiological and environmental measurements in actual driving conditions. Drivers wore Empatica E4 wristbands and Zephyr BioHarness 3 chest straps (manufactured by Zephyr Technology Corporation, Annapolis, MD, USA) which recorded physiological signals including electrodermal activity from both wrists, heart rate, breathing rate, and skin temperature. In addition to these physiological signals, the dataset includes contextual data such as GPS location, in-car temperature, humidity, sound level, and synchronized video recordings of both the vehicle interior and the external driving environment. A continuous, real-time stress metric was also recorded during each drive. This metric was annotated by an observer seated in the rear seat of the vehicle, who used a slider to indicate the perceived overall stress level on a scale from low (0) to high (1). After each driving session, the driver reviewed the video footage and validated or corrected the stress annotations to ensure their accuracy. This rich dataset is particularly beneficial for the investigation of levels of stress and attention in the participants. The class labels in our models were assigned according to the mean stress levels throughout the session: ‘no stress’ for class 0, ‘medium stress’ for class 1, and ‘high stress’ for class 2. These thresholds were initially established using the AffectiveROAD dataset and further validated by participant survey response [24].

We used the Random Forest Classifier and Gradient Boosting Classifier as two base models. For each base classifier, a randomized search strategy was applied over a predefined hyperparameter space, with 100 iterations. The Random Forest classifier was tuned across the following hyperparameters: number of estimators (n_estimators, range 50–300), maximum tree depth (max_depth, range 10–30), number of features considered at each split (max_features, either ‘sqrt’ or ‘log2’), minimum samples required to split an internal node (min_samples_split, range 2–20), minimum samples required at a leaf node (min_samples_leaf, range 1–10), and bootstrap sampling (bootstrap, either True or False). For the Gradient Boosting classifier, the tuned hyperparameters included n_estimators (range 50–300), max_depth (range 3–10), and learning_rate (continuous uniform distribution between 0.01 and 0.31). We then employed nested cross-validation using two levels of Stratified 5-Fold cross-validation. In inner cross-validation, the training dataset was split into 5 folds to optimize the hyperparameters. In outer cross-validation, the entire dataset was split into 5 folds to evaluate the model with best-performing inner cross-validation hyperparameters.

The reason why the Stratified K-Folds method was employed in this study is because the dataset was imbalanced, and this ensured that class distribution was the same in each fold. After hyperparameter tuning, the best hyperparameters were selected, and the models were trained on the training set. Their performance was then measured on the test set, using precision, recall, and F1 score metrics. The performance statistics of each base model are presented in Table 1 (for Random Forest) and Table 2 (for Gradient Boosting).

The Random Forest model performed well in nested cross-validation and testing. It averaged 96.18% accuracy in cross-validation, indicating that the model is capable of generalizing to new data. The best hyperparameters had a high number of trees (178 estimators), a deep tree structure (maximum depth of 29), and log2 for feature selection. On the test data, the model achieved 98% accuracy with high precision and recall in all classes. The precision and recall values in class 0 and class 2 were close to 1, whereas in class 1, where the lowest result was obtained, the corresponding precision and recall values were at 0.95 and 0.94.

The Gradient Boosting model also performed well, although slightly lower than the Random Forest. Its nested cross-validation scores averaged 95.35%, which reflected good performance but with greater sensitivity to parameter variation. The optimized parameters were a learning rate of 0.228 and depth of 9. In the test set, it had an overall accuracy of 97%. The performance metrics were high for all classes, particularly for classes 0 and 2. Class 1 had a lower recall of 0.91 in comparison to Random Forest but possessed high precision at 0.96.

The results of inner and outer cross-validation scores showed no overfitting. Overfitting would generally occur when a model has high performance on training data but low performance on unseen data, and this would reflect in a large gap between training and validation scores. However, the model results show consistent performance in inner and outer loops with no significant differences in scores.

In addition, a Stacking Classifier was employed to combine the Random Forest and Gradient Boosting models’ classification outcomes. Stacking is designed to take advantage of each model’s strengths, with the potential to enhance overall predictive accuracy. The stacked model also underwent an identical process of testing, and performance metrics were generated to verify its capacity to generalize to unseen data. The model achieved the best performance with cross-validation accuracy at 96.4%.

This indicated that the merging of the power of both models gave improved performance. The Stacking model also achieved an accuracy of 98% in the test set, the same as the Random Forest (as seen in Table 3). Recall, accuracy, and F1 scores across all the classes were high with high performance in class 2 and competitive scores in classes 0 and 1. This confirmed that the stacking approach worked in combining the strengths of the individual models as they complemented one another, resulting in overall better classification performance.

The selection of physiological features, such as heart rate, skin temperature, and electrodermal activity was guided by prior literature indicating their strong association with sympathetic nervous system activation and stress responses. Electrodermal activity, in particular, has been widely validated as a sensitive marker of acute stress and arousal in both laboratory and applied settings, which justified its prioritization. The machine learning models (Random Forest, Gradient Boosting, and Stacking Classifier) were selected due to their robustness, capacity to handle complex, non-linear relationships, and proven high performance in prior stress classification studies. Random Forest offers interpretability and resistance to overfitting; Gradient Boosting enhances predictive power through iterative refinement; and the Stacking Classifier capitalizes on model complementarity to improve generalization. These models align with our research objective of developing a reliable and accurate classification framework to assess stress levels from physiological data within the MR simulation.

We also evaluated several other machine learning models to ensure thorough analysis of the data. These included Logistic Regression, Support Vector Machine (SVM), K-Neighbors, and Adaptive Boosting. Table 4, Table 5, Table 6 and Table 7 display the optimal hyperparameters and classification reports for each model. Despite the capabilities of these individual algorithms, the Stacking Classifier consistently outperformed them and showed its ability to effectively combine the predictive strengths of both the Random Forest and Gradient Boosting models.

### 3.3. Features Analysis

To illustrate the impact of each feature in prediction results, feature importance scores were calculated using the Mean Decrease in Impurity (MDI) method, as implemented in the scikit-learn library. This approach quantifies the contribution of each feature to the predictive performance of the ensemble model by measuring the extent to which the feature reduces node impurity across all trees.

Specifically, the importance of a feature ƒ was calculated as the total reduction in the criterion (Gini impurity) brought by all splits on f across all trees, averaged and normalized. Formally, the feature importance I(ƒ) for feature ƒ can be represented as:

$$I(ƒ)=\frac{1}{T}\sum_{t=1}^{T}\sum_{n\in N_{f}^{t}}∆i(n)$$

where T is the total number of trees in the ensemble, $N_{f}^{t}$ denotes the set of nodes where feature ƒ was used to split in tree t, and ∆i(n) represents the impurity decrease at node n. To ensure comparability between models, raw feature importance values were then normalized such that the sum of all importances for each model equaled one. Normalization was achieved by dividing each importance score by the total sum of importances.

**Figure 4:**
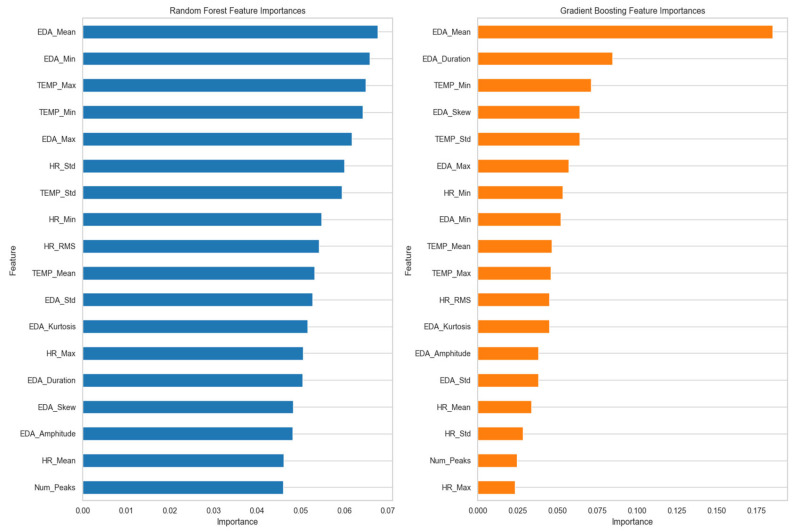
Normalized feature importance from Random Forest and Gradient Boosting models.

Figure 4 indicates the most significant features that were identified through each model. As illustrated in the figure, in the Random Forest model, the highest feature score was the mean value of EDA (EDA_Mean), which suggested that changes in EDA_Mean hold significant information about stress. Following EDA_Mean, EDA minimum value (EDA_Min) and skin temperature maximum value (TEMP_Max) were ranked second. Such features contribute to understanding the extremes and ranges of the values of EDA and temperature, which are important in assessing stress.

Skin temperature minimum value (TEMP_Min) and EDA maximum value (EDA_Max) also improved the model’s predictive ability by providing more information about temperature extremes and peak EDA values, respectively. Factors like heart rate standard deviation (HR_Std), temperature standard deviation (TEMP_Std), and heart rate minimum value (HR_Min) were of lesser importance but still played a role in capturing fluctuations related to stress intensity.

On the other hand, the Gradient Boosting model attributed more weight to EDA_Mean. EDA_Duration was also an important feature in this model, and it represents the total time that the EDA signal stayed elevated within the time window. Minimum temperature (TEMP_Min) was another important feature for Gradient Boosting; however, EDA skewness value (EDA_Skew) and EDA amplitude value (EDA_Amplitude) were features but to a lesser degree than EDA_Mean and EDA_Duration, meaning that while they were significant, their influence was slight.

In comparing both models, EDA_Mean is a significant feature in both Gradient Boosting and Random Forest but is more significant in Gradient Boosting. This discrepancy is what indicates that Gradient Boosting’s modeling approach is more capable of identifying the mean EDA value in the case of stress. The different level of significance of features like EDA_Min and TEMP_Min across the models indicated that both models utilize different data parameters. Random Forest may be more attuned to changes in these features, while Gradient Boosting may be able to detect more subtle patterns of stress.

**Figure 5:**
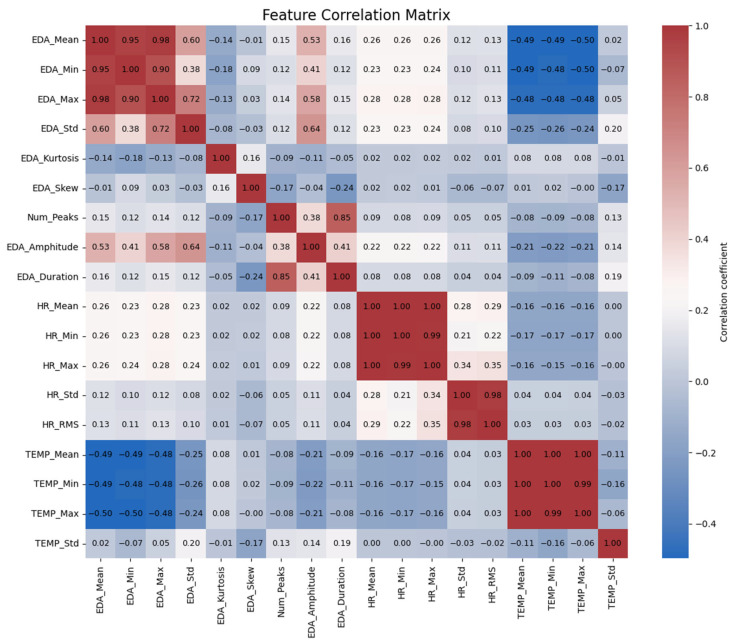
Correlation matrix of the features.

Secondly, we examined the correlation between these features presented in Figure 5. The correlation matrix indicates that all three metrics correlate which confirm that changes in one metric are related to changes in the others.

The correlation matrix further reveals interrelationships among the physiological measurements. Firstly, it shows an inverse relationship between body temperature and EDA. In other words, EDA_Mean is inversely related to TEMP_Mean (−0.49), TEMP_Min (−0.49), and TEMP_Max (−0.50). This relationship persists across the measurements of EDA, because EDA_Max is also inversely related to TEMP_Mean (−0.50). This suggests that with increases in stress levels, as indicated by EDA increases, there is an accompanying decrease in skin temperature. This inverse effect suggests a physiological phenomenon in which increased stress or arousal is accompanied by a decrease in surface temperature as vasoconstriction redirects blood to the core of the body [25].

In addition, EDA is positively correlated with heart rate. EDA_Mean is correlated with HR_Mean (0.26) and HR_Max (0.26), indicating that higher levels of EDA are associated with increased heart rates. This confirms that as individuals experience more stress, their heart rate increases. The same goes for EDA_Min, but with a poorer though still significant correlation with HR_Mean (0.23), indicating that even the lowest values of EDA are in some way associated with heart rate means.

EDA amplitude and duration also yield useful information. EDA_Amplitude is positively related to both EDA_Mean (0.53) and EDA_Max (0.58) at a moderate level, suggesting that larger fluctuation in skin conductance is associated with larger overall levels of EDA. Moreover, EDA_Duration is positively related to HR_Min (0.29), which means that longer durations of high electrodermal activity are associated with lower minimum heart rates. This could imply that long-lasting stress responses are associated with a decrease in the baseline heart rate.

Finally, relations are seen between the distribution measures of EDA, i.e., kurtosis and skewness. EDA_Kurtosis is positively correlated with EDA_Std (0.72) at high significance levels, such that increased deviation from the mean is associated with more variability in EDA. EDA_Skew, on the other hand, is not highly correlated with other variables, and this suggests that asymmetry in EDA distributions is unlikely to have a significant in-fluence on other physiological variables. These findings offer a glimpse into the intricate relationship between EDA, heart rate, and temperature and how they all react as a coordinated system to stress in the body.

## 4. Pilot Study Results

A total of nine pre-licensure nursing students and three nurse faculty members of the University of Arizona College of Nursing, aged between 20 and 49 years (mean = 29.6, SD = 10.1), volunteered to participate in our pilot study approved by the IRB. Each participant spent approximately 2 h on the experiment, yielding a total of 86,400 s of data collected. The participant demographic information is indicated in Table 8, offering a mix of levels of education and experience that reflect a diverse group of participants in the study.

**Figure 6:**
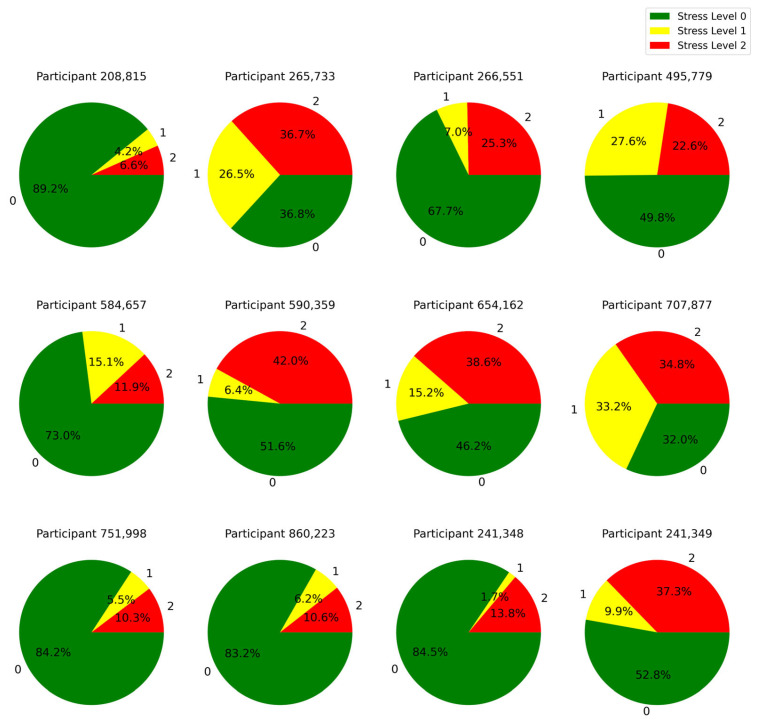
Distribution of time spent by each learner across different stress levels: 0 (low), 1 (medium), and 2 (high).

A representation of the distribution of time over different stress levels for all the subjects in our experiment is given in Figure 6. This result gives a clear view of stress level distributions across subjects by showing how much time each subject spent in each of the different stress states. Additionally, the participants’ answers to the debriefing questions at the end of the simulation are summarized in Table 9.

Based on Figure 6 and Table 9, we identified that all participants who experienced stress levels of 2 for more than 25% of the simulation time, reported that they were not successful in fully treating the patient. This highlights the point that stress level 2 during the training had an impact on the participants’ performance outcomes. This insight can be used to further personalize the training by decreasing the complexity of the simulation or offering supportive cues in the mixed reality environment when the stress reaches level 2.

### 4.1. Scenario-Based Validation

To ensure the applicability of the stress classification model to actual settings, we employed scenario-based validation within the context of the pilot study. This validation involved a critical segment of the simulation (segment 4) in which participants had to react to an emergency medicine scenario where the patient was on the verge of developing hypotension and hypoxia; situations that need prompt and effective decision-making.

The result of this validation showed that the model correctly classified all participants as being under a higher level of stress (Stress Level 1 or Stress level 2) during the emergency scenario. This consistent classification across all test populations suggests that the model is sensitive and accurate in detecting heightened stress reactions to critical, high-stakes situations.

This scenario-based validation supports both the accuracy of the model’s classifications and its practical value in real clinical settings. The model holds significant potential for use in training environments focused on understanding and managing stress, as it accurately reflects the stress responses typically seen in such situations. This can help improve clinical performance and patient outcomes.

### 4.2. Variation in Physiological Markers Across Stress Levels

**Figure 7:**
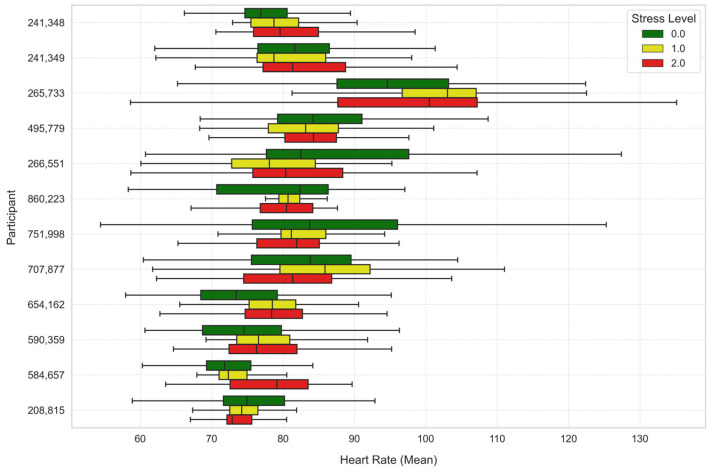
Average heart rate across stress levels for each learner.

**Figure 8:**
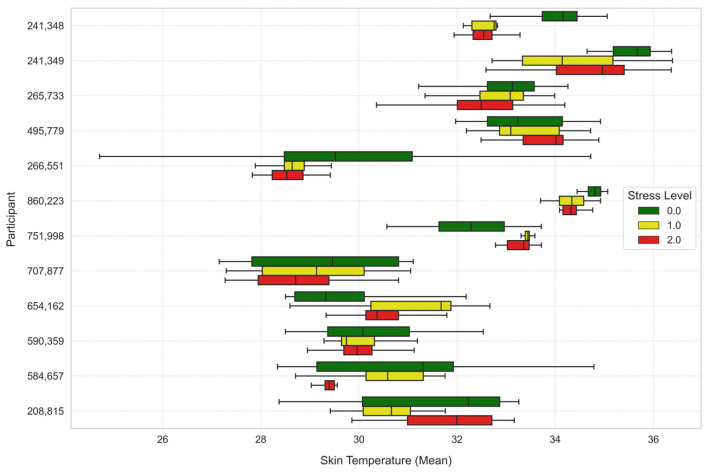
Average skin temperature across stress levels for each learner.

**Figure 9:**
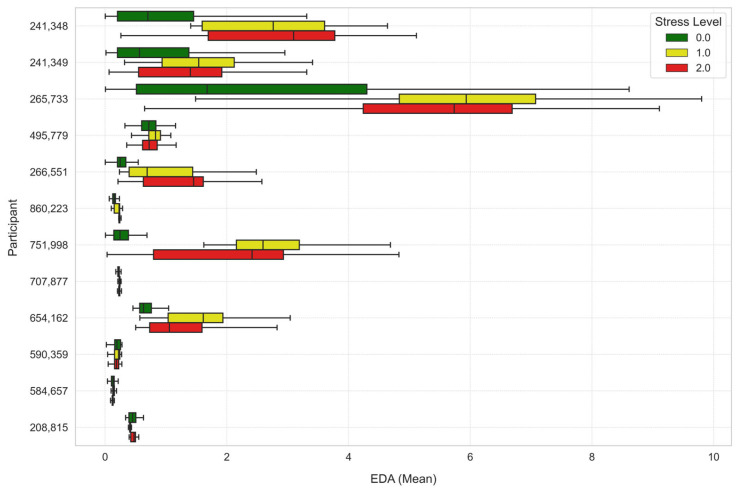
Average electrodermal activity across stress levels for each learner.

To further analyze the stress levels, Figure 7, Figure 8 and Figure 9 illustrate the way mean heart rates, skin temperature, and electrodermal activity vary amongst learners across the different stress levels.

We then used paired *t*-tests to determine differences in physiological measurements across the three levels of stress (Stress Level 0, 1, and 2). The analysis was performed over 86,400 s of data and the summaries of the results are presented in Table 10 and Table 11.

There were considerable differences in heart rate in both analyses. Under Stress Level 1, the heart rate was significantly higher compared to Stress Level 0 (t-statistic = 13.7554, *p*-value ≈ 0.0), indicating a substantial increase in heart rate with elevated stress. Similarly, the comparison of Stress Level 2 versus Stress Level 0 also showed an increase in heart rate at significance level (t-statistic = 11.0204, *p*-value ≈ 0.0).

Mean skin temperature showed significant decrease through levels of stress. Stress Level 1 recorded a lower skin temperature than Stress Level 0 (t-statistic = −6.8260, *p*-value ≈ 0.0), and the difference was even larger at Stress Level 2 (t-statistic = −14.7139, *p*-value ≈ 0.0).

EDA also recorded significant differences by levels of stress, where under Stress Level 1 was significantly higher than Stress Level 0 (t-statistic = 28.2458, *p*-value ≈ 0.0), and the same significant rise was achieved under Stress Level 2 compared to Stress Level 0 (t-statistic = 36.7045, *p*-value ≈ 0.0). The rise in EDA is reflective of heightened sympathetic nervous system activity, leading to heightened sweating and greater skin conductance due to stress.

In conclusion, the outcome of the *t*-test indicated that heart rate, skin temperature, and electrodermal activity varied significantly with the variation in stress level. In other words, such physiological measures could be reliable indicators for the assessment of stress and may aid in the design of effective stress management interventions within the mixed reality settings.

### 4.3. Participants Feedback

Participants reported that the MR simulations presented realistic and engaging clinical scenarios that elicited cognitive and emotional demands similar to real-world patient care. Many noted feeling mentally challenged when prioritizing patient needs such as fluid resuscitation, oxygen administration, and infection management. The integration of digital patients who could communicate and respond added an additional layer of realism that increased situational awareness and heightened stress, particularly when monitoring for critical risks like hypotension.

While several participants acknowledged moments of increased stress due to managing complex tasks and interpreting patient responses, they also highlighted that the simulation environment provided a safe space to practice under pressure without risking patient safety. One participant emphasized, “Keeping my patient alive was my primary focus, which made the experience intense but rewarding”. Another shared, “I was constantly watching for signs of deterioration, which kept me engaged and aware of time pressures”.

Participants consistently expressed that the MR simulations enhanced their clinical competence and decision-making skills. They appreciated the opportunity to interact with digital patients using both verbal communication and physical actions, such as administering oxygen or preparing vasopressors. Several participants stated that the experience improved their understanding of prioritizing care under dynamic clinical conditions.

Most participants rated the MR system as superior or comparable to traditional manikin-based training. They found that the interactive and immersive nature of the simulation helped solidify nursing procedures and improve communication skills with patients. One participant noted, “Compared to a manikin, this felt more realistic, and it helped me better prepare for actual patient interactions”.

## 5. Conclusions

Stress is a significant concern in the nursing field that impacts both the nurses’ health and the quality of patient care. To address the issue, we conducted an experiment aimed at measuring levels of stress in nursing students in a mixed reality training system. In the experiment, subjects were exposed to digital patients and clinical equipment, replicating real-life health environments.

The research included 2 h training per learner, divided into six segments. Learners were exposed to clinical scenarios developed to replicate high-pressure healthcare environments, and they directly interacted with the digital patients. The virtual patients had been integrated with a conversational AI model that allowed for natural-sounding voice interactions. In addition, the patient’s facial expressions were scripted to react based on their medical condition and emotional state, adding to the realism of the simulation.

Physiological signals such as heart rate, electrodermal activity, and skin temperature were continuously recorded using the Empatica E4 wristband. These measurements were employed as objective indices to assess the nurses’ stress levels during the simulation. Physiological signals were pre-processed to achieve accuracy and consistency for all measurements. Afterwards, statistical measures such as mean, standard deviation, skewness, and kurtosis were extracted for each physiological signal. Additionally, dynamic changes in the signals such as peak detection in electrodermal activity and root mean square values in heart rate and temperature were examined.

A Stacking Classifier of Gradient Boosting and Random Forest was then used to classify the participant’s level of stress. Nested cross-validation was implemented for hyperparameter tuning and model selection. The Stacking Classifier performed the best among all, which had a 98% accuracy on the test set. The model combined well the strengths of Random Forest and Gradient Boosting and achieved superior predictive power in classifying stress levels.

Our findings highlight that the high-pressured mixed reality training environment significantly impacts the physiological level of stress in nursing students. Participants displayed measurable responses to stress, with elevations in heart rates and electrodermal activity on high-stress sections of the simulation.

The findings of this study can inform the integration of MR simulations into nursing education to enhance both technical competencies and stress management skills. For example, MR scenarios replicating high-acuity patient care situations could be embedded into simulation-based courses to allow students to practice managing stress while making clinical decisions in a safe environment. Additionally, MR modules could be used as refresher training for practicing nurses in critical care or emergency settings to maintain readiness and resilience. These applications support a more experiential, self-regulated approach to developing both clinical and emotional competencies essential for high-stakes healthcare delivery.

## 6. Discussion and Future Work

Although this study identified nursing students’ stress levels in MR settings, there are certain avenues for future research that can assist in improving the understanding and application of MR in nursing education.

An important avenue for future research involves investigating the long-term impact of MR-based training on stress management and clinical performance. Although the present study focused on immediate stress responses during simulation sessions, it remains unknown whether repeated exposure to MR scenarios can enhance nurses’ resilience and stress-coping skills in real clinical environments. Longitudinal studies assessing retention of stress-management strategies and transferability to practice (potentially through follow-up assessments weeks or months post-training) would provide valuable insights into the sustained benefits of this approach. Such research could also explore whether MR-based training reduces stress-induced decision errors or improves patient outcomes in high-pressure clinical scenarios.

Another course for future work will be real-time stress detection with adaptive interventions during training. Since this study was aimed at recording physiological data, for example, heart rate, skin temperature, and electrodermal activity in order to measure the level of stress, future research could involve real-time analysis of these data with mechanisms for automated feedback. For example, adaptive systems can be programmed to offer relaxation techniques, such as guided breathing, when heightened levels of stress are detected. This real-time feedback would enhance the learning experience by enabling nursing students to manage their levels of stress more effectively, so they are buffered during stressful training exercises but still reap the benefits of the pressure that comes with realistic environments.

This would also give a more profound understanding of the stress level by varying the physiological data that is recorded. Whereas the experiment made use of heart rate, electrodermal activity, and skin temperature, future experiments may consider using other biomarkers, such as respiratory rate or brain-wave activity (EEG), thereby adding more proof of the multi-responses of the physiological systems occurring under stress in XR environments. In addition, the inclusion of these markers could increase the accuracy of stress detection, allowing for a more comprehensive view of the nurse’s physiological state during training.

Additionally, the relatively small sample size of our study limits the generalizability of the results. The participants were drawn from a specific population of nursing students within a single institution, which may not fully represent the broader diversity of practicing nurses or students from different educational backgrounds. Future research should aim to replicate and extend these findings using larger and more diverse participant groups to enhance external validity and deepen the understanding of individual differences in stress responses within extended reality environments.

Furthermore, monitoring physiological data in educational settings raises important ethical considerations that warrant careful attention. Participants’ privacy must be safeguarded through secure storage of sensor data, anonymization protocols, and clear communication about data usage. Consent procedures explicitly outlined the nature of data collection, how data would be used, and participants’ right to withdraw at any time without penalty. Additionally, continuous monitoring during simulations may introduce psychological discomfort or heightened self-awareness, potentially influencing behavior. To mitigate this, participants were thoroughly briefed and given opportunities to express concerns. Future applications of such technologies in education should prioritize transparency, minimize intrusiveness, and ensure that physiological monitoring is framed as a tool to enhance learning, not as an evaluative measure that could induce anxiety or judgment.

By addressing these areas in future research, MR-based training for nurses can be an even more powerful, more specific, and more effective way of preparing healthcare professionals to manage high-stress environments with greater resilience and effectiveness.

## Figures and Tables

**Table 1:** Random Forest test set performance.

Metric	Class 0	Class 1	Class 2
Precision	0.99	0.95	0.98
Recall	0.99	0.94	0.98
F1 Score	0.99	0.94	0.98
Support	1082	450	957
Accuracy	0.98	0.98	0.98
Macro Avg Precision	0.97	0.97	0.97
Macro Avg Recall	0.97	0.97	0.97
Macro Avg F1 Score	0.97	0.97	0.97
Macro Avg Precision	0.98	0.98	0.98
Weighted Avg Recall	0.98	0.98	0.98
Weighted Avg F1 Score	0.98	0.98	0.98

**Table 2:** Gradient Boosting test set performance.

Metric	Class 0	Class 1	Class 2
Precision	0.99	0.96	0.96
Recall	0.99	0.91	0.98
F1 Score	0.99	0.93	0.97
Support	1082	450	957
Accuracy	0.97	0.97	0.97
Macro Avg Precision	0.97	0.97	0.97
Macro Avg Recall	0.96	0.96	0.98
Macro Avg F1 Score	0.96	0.96	0.97
Macro Avg Precision	0.97	0.97	0.97
Weighted Avg Recall	0.97	0.97	0.97
Weighted Avg F1 Score	0.97	0.97	0.97

**Table 3:** Stacking classifier test set performance.

Metric	Class 0	Class 1	Class 2
Precision	0.99	0.93	0.98
Recall	0.99	0.94	0.97
F1 Score	0.99	0.94	0.98
Support	1082	450	957
Accuracy	0.98	0.98	0.98
Macro Avg Precision	0.97	0.97	0.97
Macro Avg Recall	0.97	0.97	0.97
Macro Avg F1 Score	0.97	0.97	0.97
Macro Avg Precision	0.98	0.98	0.98
Weighted Avg Recall	0.98	0.98	0.98
Weighted Avg F1 Score	0.98	0.98	0.98

**Table 4:** Logistic regression.

Metric	Value
Fitting Details	5 folds for each of 45 hyperparameter candidates, totaling 225 fits
Best Parameters	{‘C’: 0.001, ‘max iteration’: 100, ‘solver’: liblinear}
Best Accuracy	0.6105
Test Set Accuracy	0.6102
Test Set Precision	0.6180
Test Set Recall	0.4957
Test Set F1 Score	0.4513

**Table 5:** Support Vector Machine (SVM).

Metric	Value
Fitting Details	5 folds for each of 24 hyperparameter candidates, totaling 120 fits
Best Parameters	{‘C’: 100, ‘gamma’: ‘scale’, ’kernel’: radial basis function}
Best Accuracy	0.7612
Test Set Accuracy	0.7794
Test Set Precision	0.7473
Test Set Recall	0.7231
Test Set F1 Score	0.7311

**Table 6:** K-Neighbors.

Metric	Value
Fitting Details	5 folds for each of 16 hyperparameter candidates, totaling 80 fits
Best Parameters	{‘metric’: Manhattan, ‘number of neighbors’: 3, ‘weights’: distance}
Best Accuracy	0.9054
Test Set Accuracy	0.9274
Test Set Precision	0.9118
Test Set Recall	0.9113
Test Set F1 Score	0.9115

**Table 7:** Adaptive Boosting.

Metric	Value
Fitting Details	5 folds for each of 125 hyperparameter candidates, totaling 625 fits
Best Parameters	{‘base estimator max depth’: 5, ‘learning rate’: 0.1, ‘number of estimators’: 300}
Best Accuracy	0.9035
Test Set Accuracy	0.9206
Test Set Precision	0.9197
Test Set Recall	0.8954
Test Set F1 Score	0.9049

**Table 8:** Demographics of study participants.

Demographics	Percentage of Participants
Nursing Education	
Less than one semester of nursing courses	25%
One to two semesters of nursing courses	42%
Bachelor’s degree	8%
Master’s degree	25%
Experience Level	
No experience	33%
Less than 2 years	8%
2 to 5 years	17%
6 to 10 years	25%
21 to 25 years	17%
Types of healthcare simulations participated in	
Standardized patient	33%
Manikin	50%
Virtual screen-based	17%
Virtual with headset	17%
Mixed reality with headset	8%
Experience with Virtual Reality headset	8%
Very little	58%
None	50%
Experience with Mixed Reality headset	
Very little	67%
None	58%

**Table 9:** Summary of participants’ simulation goals, patient priorities, risks, required equipment, actions taken, and success.

Participant Number	Goal During Simulation	Patient’s Priority Needs	Patient’s Risks	Essential Items	Actions Taken	Succesfull?
208,815	Keeping my patient alive	Watching for hypovolemia and sepsis	Sepsis, hypovolemic shock, hemorrhage	Oxygen, Blood pressure, IV pump/suction	Recognized low blood pressure, patient unresponsive, called rapid response, initiated protocols	Yes
265,733	Contribute to education and patient survival	Blood pressure, perfusion to major organs	Loss of oxygenation, internal bleeding	IV fluids, Oxygen, electrolytes	Adjusted IV rate, monitored vitals, called rapid response, bolus of Lactated Ringer’s, 4L O_2_	No
266,551	Becoming oriented with virtual tools	Assessing the patient	Impaired gastric motility, altered bowel habits, pressure injury	Oxygen, call light, Blood pressure machine	Thorough assessment, evaluated chart before engaging	No
495,779	Improve patient assessment skills	Follow doctors’ orders, monitor vitals	Pulmonary embolism, low O_2_, hypertension	Ambu bag, supplemental O_2_, code cart	Assessed patient, applied oxygen	Yes
584,657	Learn AR benefits for nursing education	Pain and infection control	Sepsis, severe pain, malnutrition	Ambu bag, IV, monitors	Monitored vitals, IV fluids, assessment, used call light, reviewed orders	Yes
590,359	Checking vitals, administering IV, oxygen	Oxygen, IV	Blood infection, sepsis, hypotension	Oxygen, IVs, blood glucose	Used oxygen mask, administered IV	No
654,162	Implement interventions, monitor vitals	Oxygen, NG suction, IV bolus, vital sign observation	Hypotension, hypoxia, pain	Oxygen, suction, IV bolus	Adjusted IV rate, turned on O_2_/suction, administered bolus, notified MD, monitored vitals	No
707,877	Provide competent care in a safe environment	Addressing safety errors, pain management, responding to sepsis	Cardiac arrhythmias, organ failure, death	Ambu bag, oxygen source, code cart	Verified call light, placed patient on suction, changed IVF rate, called rapid response, followed protocol	No
751,998	Learn MR, use HoloLens, navigate space	Fluid resuscitation, oxygen, antibiotics	Septic shock, severe hypotension, hypoxemia	Pressure bag, surgical team, vasopressors	Locked bed, raised side rails, administered fluids, called rapid response, updated provider, assessed for deterioration	Yes
860,223	Maintain patient’s O_2_, ensure breathing, contact provider	Oxygen, chest pain, circulation	Heart attack, low O_2_, circulation loss	EKG, heart shock kit, CPR equipment	Increased oxygen, positioned patient upright, monitored BP	No
241,348	Practice nursing and critical thinking	Oxygenation, monitoring chest pain, breathing pattern, BP	Myocardial infarction, pulmonary embolism, stroke	Oxygen, IV site, AED	Administered oxygen, constant monitoring, called rapid response and provider	Yes
241,349	Follow hypotension protocol, manage NG tube suction	Stabilizing vitals	Infection, low O_2_, hypotension	Oxygen, suction, code cart	Lowered bed, monitored vitals, called rapid response and doctor, applied oxygen, allowed family access	No

**Table 10:** Mean comparison results.

Metric	Stress Level 0	Stress Level 1	Stress Level 2
Heart Rate Mean	82.23	86.30	84.79
Skin Tempreture Mean	31.81	31.49	31.23
Electrodermal Activity Mean	0.68	2.09	1.87

**Table 11:** T-test results.

Comparison	T-Statistics	*p*-Value	Significance
Stress Level 1 vs. Stress Level 0 (HR_Mean)	13.7554	*p* < 0.001	Statistically significant
Stress Level 2 vs. Stress Level 0 (HR_Mean)	11.0204	*p* < 0.001	Statistically significant
Stress Level 1 vs. Stress Level 0 (TEMP_Mean)	−6.8260	*p* < 0.001	Statistically significant
Stress Level 2 vs. Stress Level 0 (TEMP_Mean)	−14.7139	*p* < 0.001	Statistically significant
Stress Level 1 vs. Stress Level 0 (EDA_Mean)	28.2458	*p* < 0.001	Statistically significant
Stress Level 2 vs. Stress Level 0 (EDA_Mean)	36.7045	*p* < 0.001	Statistically significant

## Data Availability

The data supporting the findings of the study is not publicly available but can be accessed by contacting the corresponding author directly.
